# Use of provider-to-provider telemedicine in Kenya during the COVID-19 pandemic

**DOI:** 10.3389/fpubh.2022.1028999

**Published:** 2022-11-21

**Authors:** Erin J. Kim, Meghan E. Moretti, Antony Mugambi Kimathi, Stephen Y. Chan, Richard Wootton

**Affiliations:** ^1^The Addis Clinic Inc., Nashville, TN, United States; ^2^Division of Cardiology, Department of Medicine, Pittsburgh Heart, Lung, Blood, and Vascular Medicine Institute, University of Pittsburgh School of Medicine and University of Pittsburgh Medical Center, Pittsburgh, PA, United States; ^3^Collegium Telemedicus, Taunton, United Kingdom

**Keywords:** digital health, telemedicine, coronavirus, COVID-19 pandemic, frontline health workers, LMICs, health systems

## Abstract

**Introduction:**

According to the World Health Organization (WHO), about 90 percent of countries continue to report COVID-related disruptions to their health systems. The use of telemedicine has been especially common among high-income countries to safely deliver and access health services where enabling infrastructure like broadband connectivity is more widely available than low- and middle-income countries (LMICs). The Addis Clinic implements a provider-to-provider (P2P) asynchronous telemedicine model in Kenya. We sought to examine the use of the P2P telemedicine platform during the second year of COVID-19.

**Methods:**

To assess sustainability, we compared the data for two 12-month calendar periods (period A = year 2020, and period B = year 2021). To examine performance, we compared the data for two different 12-month periods (period C = pandemic period of February 2021 to January 2022, and period D = baseline period of February 2019 to January 2020).

**Results:**

Sustainability of the P2P telemedicine platform was maintained during the pandemic with increased activity levels from 2,604 cases in 2020 to 3,525 cases in 2021. There was an average of 82 specialists and 5.9 coordinators during 2020, and an average of 81 specialists and 6.0 coordinators during 2021. During 2020, there were 444 cases per coordinator, and 587 cases per coordinator in 2021(*P* = 0.078). During 2020, there were 32 cases per specialist, and 43 cases per specialist in 2021(*P* = 0.068). Performance decreased with 99 percent of cases flagged as “answered” during the baseline period (period D), and 75 percent of cases flagged as “answered” during the pandemic period (period C).

**Conclusion:**

Results suggest that despite a decline in certain sustainability and performance indicators, The Addis Clinic was able to sustain a very high level of activity during the second year of the pandemic, as shown by the continued use of the system. Furthermore, despite some of the infrastructure challenges present in LMICs, the P2P telemedicine platform was a viable option for receiving clinical recommendations from medical experts located remotely. As health systems in LMICs grapple with the effects of the pandemic, it is worthwhile to consider the use of telemedicine to deliver essential health services.

## Introduction

According to the World Health Organization (WHO), about 90 percent of countries continue to report COVID-related disruptions to their health systems, with 66 percent citing health workforce-related issues as the most common causes (1). Reasons such as essential health workers needing to stay at home to care for sick family members, employees requiring quarantine because of COVID exposure, and health professionals leaving the workforce have put extreme pressure on health systems. Moreover, the pandemic has diverted many of the resources for health away from other areas. A recent report by The Global Fund suggests that the spillover effects of COVID-19 have eroded decades of progress in fighting diseases such as human immunodeficiency virus (HIV), tuberculosis (TB), and malaria (2). COVID-related disruptions have been particularly harmful for low- and middle-income countries (LMICs) due to the unpredictable nature of the disruptions coupled with the evolving pandemic. Furthermore, public health measures like social distancing and patients' fear of contracting COVID-19 have further exacerbated the decline in access to health services.

Adoption of digitally-enabled solutions like telemedicine has increased substantially since the start of the pandemic (3). Being able to digitally deliver healthcare *via* telemedicine has allowed providers and patients to safely deliver and access essential health services. Survey results from the WHO suggest that 48 percent of countries employed telemedicine to replace in-person consultations during the pandemic (1). The use of telemedicine to mitigate COVID-related disruptions has been especially common among high-income countries where enabling infrastructure like broadband connectivity is more widely available than in LMICs (4). Studies suggest, however, that there is a willingness to adopt telemedicine in low resource settings with several examples of telemedicine use during the COVID-19 pandemic (5, 6).

Kenya confirmed its first case of COVID-19 in March 2020. Since then, the country has experienced several waves of infection, with 333,000 confirmed cases up to June 2022.^1^ To curb the rate of transmission, Kenya enforced strict public health measures such as social distancing, travel restrictions, and curfews. The restricted mobility along with COVID-related fears and stigma, reduced patients' willingness to seek essential health services, and limited their access to health facilities. One study found that there was a decrease in expectant mothers choosing to deliver in health facilities because of fears of getting infected, which in turn led to an increase in home deliveries (7).

The Addis Clinic, a non-profit organization based in the United States, implements a provider-to-provider (P2P) telemedicine program in Kenya. Since 2011, The Addis Clinic has been providing access to specialized medical experts for frontline health workers (FHWs) treating patients in low resource settings. The organization connects 430 FHWs in Kenya with a network of 117 physician specialists (specialists) located remotely, providing a mechanism for the communication of diagnostic and management recommendations *via* asynchronous technology (8). The teleconsultation process begins with FHWs submitting cases to the telemedicine platform using a mobile phone app. Cases are then triaged to the appropriate specialist by in-country case coordinators (coordinators), at which point an asynchronous communication is established between the parties.

Evidence suggests that telemedicine has the potential to mitigate the spread of infectious diseases and improve access to health services in LMICs (9). Yet, most of the telemedicine reported has required enabling infrastructure, which is often a barrier in limited resource settings (9). A previous study of The Addis Clinic telemedicine work in Kenya showed that teleconsultations increased substantially during the first year of the pandemic, probably because the network had expanded its referral base by increasing the number of FHWs in Kenya (10). However, it was unclear whether the high caseloads being managed by coordinators and specialists, would be sustainable in the longer term. This is especially relevant as COVID-related disruptions exacerbated many of the systems-related issues that LMICs were already struggling with prior to the pandemic.

In the present study, we examined The Addis Clinic telemedicine work in Kenya during the second year of the pandemic to assess the sustainability of its operations. We also reviewed the performance and quality of service delivered both before and during the pandemic.

## Methods

Sustainability was measured in terms of the performance of The Addis Clinic telemedicine network. Measurement of performance is a fundamental aspect of network evaluation, and we have previously suggested a general framework for this purpose (11). The framework entails assessment of various metrics including network activity and efficiency.

### Network activity and efficiency

Network activity was measured as the number of cases submitted to the network each day, excluding any test cases.

Network efficiency can be defined as the ratio of Output to Input, where Output is a measure of what has been produced by the network, and Input is a measure of the resources that were consumed to produce that output. The output produced by a network is related to the number of cases dealt with during the period of interest, i.e., cases answered. It is also related to their complexity and to the speed of the responses provided. A crude estimate of the output can be obtained from the referral rate (10).

The input to a network is related to the resources consumed during the period of interest, an important part of which is the number of people who were needed to run the network and the time they spent doing it. We therefore considered the numbers of coordinators and the specialists who were involved in the cases that were handled during the period of interest. For these two types of users, a measure is required of the time spent in dealing with the cases. Such data is not normally available in any telemedicine network, so we estimated it from the number of coordinators and specialists who were known to be active during the period of interest.

### Network performance and service quality

Network performance was based on four simple performance statistics that are of interest to the users and the operators of a network:

- Referral rate—The number of referrals received in unit time;
- Allocation delay—The interval between the arrival of the case and the first time it is allocated for reply;
- Query delay—The time between a case being assigned to a specific specialist and that specialist responding; and
- Answer delay—The delay after a case has been submitted before the first reply is received from a specialist.

Service quality was based on the speed of the telemedicine responses provided by the network and their quality. Speed of response can be measured as the answer delay. The quality of the responses is more difficult to measure, but can be inferred from the user feedback (12): in the telemedicine system used by The Addis Clinic, requests are sent automatically to FHWs to complete a user feedback questionnaire 21 days after each teleconsultation has been submitted. FHWs can respond by completing a questionnaire containing 12 questions. The present work considered the following questions relating to service quality:

**Q1** “Was the case sent to an appropriate expert?”
**Q2** “Was the answer provided sufficiently quickly?”
**Q3**“Was the answer well-adapted for your local environment?”
**Q6** “Did you find the advice helpful?”
**Q7b** “Did it [the advice] assist with your management of the patient?”
**Q8** “Do you think the eventual outcome for the patient will be beneficial?”
**Q9** “Was there any educational benefit to you in the reply?”

Answers could be chosen from multiple choice responses (yes/no/don't know).

### Analysis

**Figure 1:**
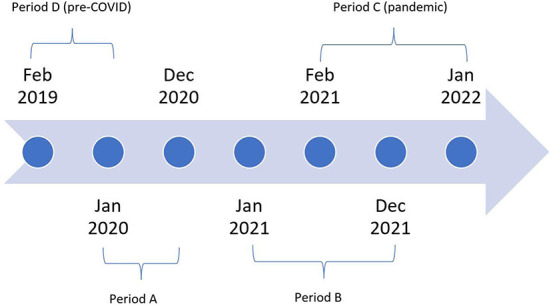
Study timeline: Period A (calendar year 2020), Period B (calendar year 2021), Period C (pandemic period = February 2021 to January 2022), and Period D (pre-COVID = February 2019 to January 2020).

We conducted a retrospective analysis of the telemedicine cases referred to The Addis Clinic from Kenya. To assess sustainability, we compared the data for two 12-month periods (period A = calendar year 2020 and period B = calendar year 2021). To assess performance, we compared the data for two 12-month periods. Period C includes the pandemic period of February 2021 to January 2022, while period D (pre-COVID) consists of the baseline period of February 2019 to January 2020, see Figure 1.

In comparing data from different study periods, differences in proportions were examined using the chi-squared test. Differences in numbers of observations were examined using the *t*-test or Mann-Whitney test, according to whether the distributions were normal or not.

Information relating to all cases was extracted from the database of the telemedicine system. Formal research ethics permission was not required, because patient consent to access the data had been obtained and the work was a retrospective chart review conducted by the organization's staff in accordance with its research policies.

## Results

### Sustainability

A comparison of data for periods A and B was used to assess sustainability.

#### Activity

**Figure 2:**
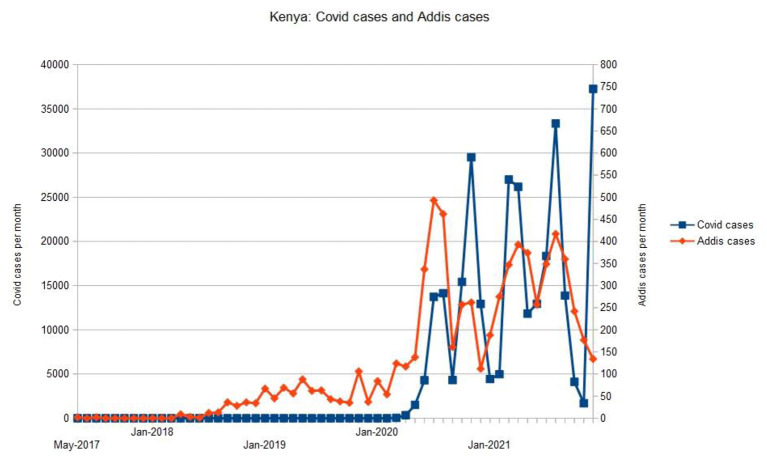
Relationship between the number of telemedicine cases referred to The Addis Clinic and the number of confirmed COVID-cases nationally.

During 2020, 2,604 cases were received; the mean submission rate was 217 cases/month (SD 147). During 2021, 3,525 cases were received; the mean submission rate was 294 cases/month (SD 94). The increase in mean submission rate was not significant (*t* = 1.52, *P* = 0.14). Changes in the number of cases referred to The Addis Clinic were associated with changes in the number of confirmed COVID-cases nationally, see Figure 2.

#### Efficiency

There was an average of 82 specialists and 5.9 coordinators during 2020. There was an average of 81 specialists and 6.0 coordinators during 2021.

During 2020, there were 444 cases per coordinator, and during 2021 there were 587 cases per coordinator. The increase was not significant (*P* = 0.078). During 2020, there were 32 cases per specialist, and during 2021 there were 43 cases per specialist. The increase was not significant (*P* = 0.068).

### Performance

A comparison of data for periods C and D was used to assess performance.

During the baseline period, 726 cases were received; during the pandemic period, 3,548 cases were received. Cases were flagged automatically as “answered” when a response was received from one or more specialists. During the baseline period, 99 percent of cases were flagged as “answered;” during the pandemic period, 75 percent of cases were flagged as “answered.”

A random sample of cases (*n* = 20) which had not been flagged as “answered” were examined. This showed that in 25 percent of these cases, the coordinator had provided advice or guidance directly to the FHW, i.e., the case had in fact been answered from the FHW's perspective. In 35 percent of cases, further information had been requested from the FHW, but not received. The remainder reflected miscellaneous other reasons for a non-answer, such as miscommunication between FHW and coordinator. The analysis which follows was conducted on the cases that had been flagged automatically as “answered.”

### Patient characteristics

The median age of the patients was 30 years (*n* = 715) during the baseline period and 28 years (*n* = 2,636) during the pandemic period. The proportions of child and adult patients were almost identical during the two study periods: 28 percent of cases were children. The median bodyweights were 60 kg (*n* = 698) and 61 kg (*n* = 1,963) during the baseline and pandemic period, respectively. The sex ratio (M:F) was 0.42 (*n* = 708) during the baseline period and 0.41 (*n* = 2,623) during the pandemic period, see Table 1.

**Table 1:** Patient characteristics.

	**Baseline**			**Pandemic**	
	***n***	**%**		***n***	**%**	
Female	411	57.1		1,536	58.1
Male	297	41.3		1,087	41.1
Not recorded	12	1.7		21	0.8
Adults (18 years and older)	511	71.0		1,884	71.3
Children (under 18 years)	204	28.3		752	28.4
Age not recorded	5	0.7		8	0.3
	**Median**	**IQR**	***n***	**Median**	**IQR**	**n**
Age (years)	30.0	16.0–43.0	715	28.0	15.0–40.0	2,636
Bodyweight (kg)	60.0	42.3–70.0	698	61.0	36.0–70.0	1,963

### Reason for referral

**Figure 3:**
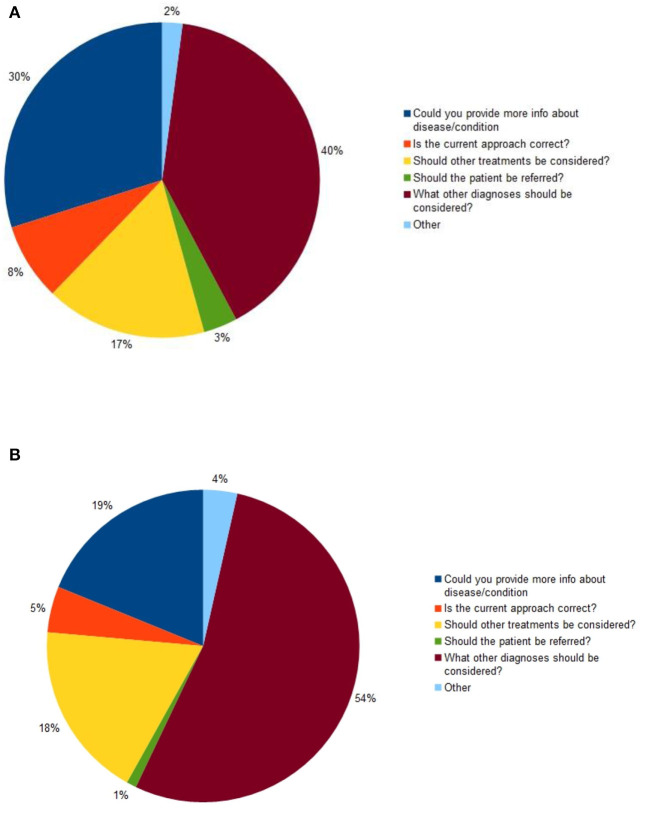
Primary reason for referral: **(A)** during the baseline period (*n* = 720); **(B)** during the pandemic period (*n* = 2,644).

The two most common reasons given for referral were “*What other diagnoses should be considered?*” and “*Could you provide more information about the disease/condition?*”. During the baseline period, these accounted for 70 percent of cases, and during the pandemic period, they accounted for 72 percent of cases, see Figure 3.

The most common reason for referral during both study periods was “*What other diagnoses should be considered?*”. During the baseline period, this was given as the reason for referral in 40 percent of cases, while during the pandemic period, it was given as the reason in 54 percent of cases: the difference was significant (chi^2^ = 40.8, *P* < 0.0001).

### Speed of response

Following the submission of a case, the median time to send it to an appropriate specialist (the allocation delay) was 1.8 h during the baseline period (*n* = 720) and 4.0 h during the pandemic (*n* = 2,444). The median time before a specialist responded (the answer delay) was 13.1 h during the baseline period (*n* = 719) and 18.9 h during the pandemic (*n* = 2,440). Seventy-eight percent of cases were answered within 48 h of submission during the baseline period (*n* = 564) and 70 percent during the pandemic (*n* = 1,838), see Table 2.

**Table 2:** Speed of response.

	**Baseline**			**Pandemic**	
	**Median**	**IQR**	***n***	**Median**	**IQR**	**n**	***z***
Allocation delay (h)^*^	1.8	0.5–4.4	720	4.0	1.4–11.4	2,444	14.2
Answer delay (h)*	13.1	5.7–39.5	719	18.9	7.4–46.7	2,440	5.2
	***n***	**%**		***n***	**%**		**chi** ^**2**^
Cases answered within 48h*	564	78.3		1,838	69.5		21.5

* The statistics exclude ~200 cases that the case coordinators answered without needing to involve a physician specialist.

### Type of expertise

**Figure 4:**
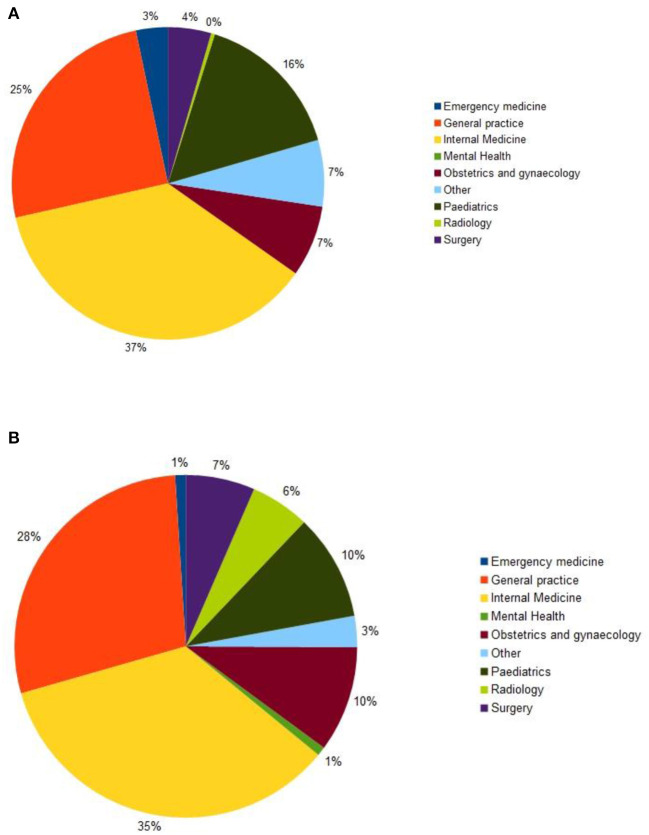
Type of expertise: **(A)** during the baseline period (*n* = 785); **(B)** during the pandemic period (*n* = 2,990).

The most common types of specialist consulted during the study were from Internal Medicine: 37 percent of cases during the baseline period (*n* = 288) and 35 percent during the pandemic (*n* = 1,036). There were minor differences between the two periods: fewer pediatricians were consulted during the pandemic period, and more radiologists, see Figure 4.

### Complexity of cases

The mean number of queries (i.e., requests to specialists) per case was 1.3 during the baseline period (*n* = 909) and 1.2 during the pandemic (*n* = 3,079). The mean number of messages about each case was 6.0 during the baseline period (*n* = 4,304) and 4.9 during the pandemic (*n* = 12,911). The median length of time between the first message about a case and the last message (the dialogue time) was 34 h during the baseline period (*n* = 719) and 24 h during the pandemic (*n* = 2,512), see Table 3. The differences were significant, see Table 3.

**Table 3:** Complexity of cases.

	**Baseline**			**Pandemic**	
	**Mean**	**SD**	***n***	**Mean**	**SD**	***n***	***z***
Queries per case	1.3	0.49	909	1.2	1.3	3,078	14.0
Messages per case	6.0	2.1	4,304	4.9	2.1	12,911	17.7
	**Median**	**IQR**	***n***	**Median**	**IQR**	***n***	***z***
Dialogue time (h)	34.0	12.8–87.9	719	23.9	9.2–63.5	2,512	4.9

### Follow-up reports

A total of 190 follow-up reports were provided by the FHWs during the baseline period, and a total of 158 reports during the pandemic. The responses to the seven questions about the value of the service were mainly positive, both at baseline and during the pandemic. For each question, the proportion of positive responses was lower during the pandemic period, and this difference was significant, see Table 4.

**Table 4:** Follow-up reports.

	**Baseline**		**Pandemic**	
	**No of responses**	**Yes %**	**No of responses**	**Yes %**	**chi^2^**
Q1 “Was the case sent to an appropriate expert?”	190	99.5	158	82.9	31.9
Q2 “Was the answer provided sufficiently quickly?”	190	98.4	158	81.0	30.4
Q3 “Was the answer well-adapted for your local environment?”	190	99.5	158	81.0	36.1
Q6 “Did you find the advice helpful?”	190	100	158	84.8	30.9
Q7b “Did it [the advice] assist with your management of the patient ?”	190	96.8	158	75.3	35.8
Q8 “Do you think the eventual outcome for the patient will be beneficial?”	190	94.7	158	76.0	25.5
Q9 “Was there any educational benefit to you in the reply?”	190	99.5	158	80.4	37.5

## Discussion

The present study shows that The Addis Clinic telemedicine platform was heavily used during both the first and second year of COVID-19. While many of the network performance indicators decreased somewhat during the pandemic period, the high volume of cases indicates that the FHWs continued to find the telemedicine service useful to them. This also suggests that despite some of the infrastructure challenges present in LMICs, the P2P telemedicine platform was a viable option for receiving clinical recommendations from medical experts located remotely. Moreover, it is notable that changes in the number of cases referred to The Addis Clinic were associated with changes in the number of confirmed COVID-cases nationally, see Figure 2. Whether patients were being redirected from overstretched public health facilities or redirected for other COVID-related reasons, the increase in the number of teleconsultations may have been a consequence of the COVID-related disruptions to the health system. It is worth mentioning that prior to COVID-19, The Addis Clinic expanded its telemedicine operation in Kenya by hiring healthcare recruiters to provide peer-to-peer training and support in the use of the telemedicine platform, as well as recruiting new FHWs to participate in the program. Additionally, the organization transitioned the responsibilities for triaging cases from U.S.-based coordinators to in-country staff to reduce logistical barriers such as time zone differences. How far these operational changes or the COVID-related disruptions to the health system contributed to the increase in teleconsultations is not known. However, our findings demonstrate that The Addis Clinic network was able to sustain a high level of activity and utilization throughout 2020 and 2021. This also suggests that if additional resources had been available, the observed falls in some quality indicators (e.g., response time) might have been avoidable.

### Network activity and efficiency

The results indicate that The Addis Clinic was operating at a high level of activity during the pandemic, with a five-fold increase in the number of teleconsultations during the pandemic compared to pre-pandemic. There were also increases in network efficiency, as judged by the numbers of cases managed by coordinators and specialists (although the increases were not significant). However, the allocation process and speed of response from specialists was slower during COVID-19 compared to the baseline. Although the number of FHWs increased during the pandemic, the number of coordinators and specialists remained the same. Nonetheless, a similar proportion of cases were answered within 48 h of submission, with 70 percent during COVID-19 compared to 78 percent during the baseline period. This suggests that The Addis Clinic network was able to manage and maintain acceptable operational standards, as it did before the pandemic. Given the environmental factors (e.g., COVID-19) during 2020 and 2021, it is fair to assume that COVID-related disruptions affecting health workers also adversely affected specialists' abilities to respond to teleconsultations, similar to reports from other health systems during the pandemic (13). It is also reasonable to infer that elements of The Addis Clinic telemedicine model (e.g., teleconsultation process, asynchronous technology), may have contributed to its resiliency during COVID-19. Operational changes such as expanding its presence in country may have allowed The Addis Clinic to absorb the increase in the level of activity from its FHWs, as evidenced by the non-significant increase in the number of cases per coordinator and the number of cases per specialist from 2020 to 2021. As future telemedicine programs are developed and deployed in LMICs, decision-makers should therefore consider establishing a strong in-country presence to ensure not only sustainability, but also resiliency during times of increased stress.

### Network performance and service quality

During the pandemic, there was a decrease in certain service quality and efficiency-related indicators (i.e., speed of response). However, The Addis Clinic was able to satisfy the needs of FHWs, as shown by the increase in activity during 2021. Moreover, the reasons for the referrals were commonly related to understanding whether other diagnoses should be considered. This suggests that the types of patients presenting to The Addis Clinic network may have had symptoms that overlapped with several other diseases, like COVID-19 and other respiratory conditions (i.e., malaria). It is also worth noting that the mean number of queries per case and the mean number of messages about each case both significantly decreased from baseline to the pandemic period. This may have been the result of FHWs improving their clinical knowledge using the telemedicine platform, resulting in less dialogue between FHW and specialist.

### Limitations

The study results are based on a single organization, which has the potential to incur bias and limit generalizability. Furthermore, it is difficult to know whether COVID-related disruptions to the health system had a spillover effect on The Addis Clinic network. Similarly, it is unclear whether operational changes made prior to the pandemic contributed to the rise in teleconsultations in 2020 and 2021. Future studies of the referral activity post-pandemic would be useful to better understand the drivers for submitting cases to the telemedicine system.

## Conclusion

Although certain service quality indicators declined during COVID-19, the present study shows that The Addis Clinic was able to sustain a very high level of activity and efficiency during the second year of the pandemic. By examining the level of activity and operational aspects of the organization delivering P2P telemedicine services, our results reveal key elements (i.e., in-country presence, asynchronous technology) needed to successfully implement such programs in LMICs. As health systems in LMICs grapple with the effects of the pandemic, it may be worth considering the use of telemedicine to deliver essential health services.

## Data availability statement

The raw data supporting the conclusions of this article will be made available by the authors, without undue reservation.

## Author contributions

EK contributed to the study design, data interpretation, and wrote the first draft of the paper. RW contributed to the study design, conducted the data analysis, data interpretation, and edited the manuscript after the first draft. MM, AK, and SC reviewed the final version and provided content input. All authors have read and agreed to the published version of the manuscript.

## Conflict of interest

Author EK served as an independent consultant, while authors MM and AK were employed by The Addis Clinic Inc. Author RW was a member of Collegium Telemedicus. Author SC has served as a consultant for Acceleron Pharma and United Therapeutics, held research grants from Actelion, Bayer, and Pfizer, was a director, officer, and shareholder of Synhale Therapeutics, and filed patents regarding metabolic dysregulation in pulmonary hypertension.

## Publisher's note

All claims expressed in this article are solely those of the authors and do not necessarily represent those of their affiliated organizations, or those of the publisher, the editors and the reviewers. Any product that may be evaluated in this article, or claim that may be made by its manufacturer, is not guaranteed or endorsed by the publisher.

## Footnotes

^1^ https://covid19.who.int/region/afro/country/ke
